# BALB/c Mice Deficient in CD4^+^ T Cell IL-4Rα Expression Control *Leishmania mexicana* Load although Female but Not Male Mice Develop a Healer Phenotype

**DOI:** 10.1371/journal.pntd.0000930

**Published:** 2011-01-04

**Authors:** Karen J. Bryson, Owain R. Millington, Thabang Mokgethi, H. Adrienne McGachy, Frank Brombacher, James Alexander

**Affiliations:** 1 Strathclyde Institute of Pharmacy and Biomedical Sciences, University of Strathclyde, Glasgow, United Kingdom; 2 Institute of Infectious Diseases and Molecular Medicine, Health Science Faculty, University of Cape Town, Cape Town, South Africa; Institut Pasteur, France

## Abstract

Immunologically intact BALB/c mice infected with *Leishmania mexicana* develop non-healing progressively growing lesions associated with a biased Th2 response while similarly infected IL-4Rα-deficient mice fail to develop lesions and develop a robust Th1 response. In order to determine the functional target(s) for IL-4/IL-13 inducing non-healing disease, the course of *L. mexicana* infection was monitored in mice lacking IL-4Rα expression in specific cellular compartments. A deficiency of IL-4Rα expression on macrophages/neutrophils (in LysM^cre^IL-4Rα^−/lox^ animals) had minimal effect on the outcome of *L. mexicana* infection compared with control (IL-4Rα^−/flox^) mice. In contrast, CD4^+^ T cell specific (Lck^cre^IL-4Rα^−/lox^) IL-4Rα^−/−^ mice infected with *L. mexicana* developed small lesions, which subsequently healed in female mice, but persisted in adult male mice. While a strong Th1 response was manifest in both male and female CD4^+^ T cell specific IL-4Rα^−/−^ mice infected with *L. mexicana*, induction of IL-4 was manifest in males but not females, independently of CD4^+^ T cell IL-4 responsiveness. Similar results were obtained using pan-T cell specific (iLck^cre^IL-4Rα^−/lox^) IL-4Rα^−/−^ mice. Collectively these data demonstrate that upon infection with *L. mexicana*, initial lesion growth in BALB/c mice is dependent on non-T cell population(s) responsive to IL-4/IL-13 while progressive infection is dependent on CD4^+^ T cells responsive to IL-4.

## Author Summary

*Leishmania* species are parasites, transmitted by sandflies which are of extensive public health importance in the tropical and subtropical regions of the world. A large number of distinct *Leishmania* species cause cutaneous disease and the vast majority of studies utilize the caustive agent of Old World cutaneous leishmaniasis, *L. major*. Other species, for example, *L. mexicana*, are associated with quite different patterns of disease following infection of mice when compared with *L. major*. Thus, while susceptible BALB/c mice deficient in the ability to respond to the cytokines IL-4/IL-13 are not protected against development of cutaneous leishmaniasis caused by *L. major* they are totally resistant to infection with *L. mexicana*. Here we describe the outcome of *L. mexicana* infection in BALB/c mice with cell-specific deletion of the receptor for IL-4/IL-13 on macrophages/neutrophils or T helper cells. Infections develop in both mutants but lesion growth is controlled only in T cell specific knockouts and female but not male mice heal. Male but not female T cell specific knockouts maintain a strong IL-4/IL-13 response. This highlights the role of IL4/IL-13 in driving a non-healing response and may in part explain why human males are more susceptible to this infection than females.

## Introduction

New world cutaneous leishmaniasis resulting from infection with *Leishmania mexicana* is under different genetic and immunoregulatory controls to those controlling *L. major* infection [1]. Also, unlike *L. major*, the majority of mouse strains are susceptible to *L. mexicana* infection [2]–[3]. As with the other *Leishmania* species, protective immunity against *L. mexicana* is the result of a STAT-4 dependent type-1 immune response, although this can be generated independently of IL-12 [4]. While the immunological pathways resulting in non-healing *L. major* infections in susceptible BALB/c mice remain somewhat controversial, IL-4 plays the major role in promoting non-healing *L. mexicana* infections in this mouse strain [5]–[7]. Thus, mice lacking IL-4 develop small lesions that heal while those lacking IL-4Rα fail to develop lesions [6]. This also indicates some input from IL-13 in the non-healing response to *L. mexicana* infection as IL-4 and IL-13 receptors share the IL-4Rα sub unit [6]. However, IL-4 and IL-13 are pleiotropic cytokines and numerous cell types of both the innate and adaptive immune responses produce these cytokines as well as express their receptors.

In order to better differentiate both the cellular sources and targets of IL-4/IL-13 initiating lesion growth and facilitating progressive non-healing disease, we have previously examined parasite growth in SCID mice reconstituted with IL-4^−/−^, IL-4Rα^−/−^, or wild type splenocytes [5]–[6]. These studies indicated that non-lymphocyte sources of IL-4/IL-13 may contribute to early lesion growth during *L. mexicana* infection. However, the non-healing disease phenotype was dependent on a lymphocyte source of IL-4 and, in its absence, IL-4-deficient splenocyte-reconstituted SCID mice generated a healing response [5]. In addition, SCID mice reconstituted with IL-4Rα^−/−^ splenocytes demonstrated that initial lesion development was also dependent upon cells from this source responding to IL-4/IL-13 [6].

In order to better differentiate the specific role of IL-4/IL-13 responding cells from global effects *in vivo*, tissue specific IL-4Rα^−/−^ mice have been produced. So far macrophage/neutrophil specific (LysM^cre^IL-4Rα^−/lox^) [8] and CD4^+^ T cell specific (Lck^cre^IL-4Rα^−/lox^) [9] IL-4Rα^−/−^ mice have been generated and the consequences for *L. major* infection studied. In contrast to susceptible BALB/c mice, BALB/c LysM^cre^IL-4Rα^−/lox^ mice showed a significantly delayed disease progression after infection with *L. major*, concomitant with normal Th2 and type 2 antibody immune responses but with improved macrophage leishmanicidal activities [8]. These results suggest that alternatively activated macrophages were contributing to the susceptible phenotype in non-healer BALB/c mice. Furthermore T cell-specific Lck^cre^IL-4Rα^−/lox^ BALB/c mice infected with *L. major* were significantly more resistant than global IL-4Rα^−/−^ mice and developed a disease phenotype and clinical immunity similar to genetically resistant C57BL/6 mice [9]; not only showing the importance of IL-4Rα signaling via CD4^+^ T cells in the non-healing BALB/c phenotype but paradoxically indicating a protective role for IL-4Rα signaling in a non-CD4^+^ T cell population.

In the present study we demonstrate that in contrast to *L. major* infection [8], macrophage/neutrophil signaling via IL-4Rα has minimal effect on the outcome of *L. mexicana* infection in BALB/c mice. In addition, unlike global IL-4Rα^−/−^ mice infected with *L. mexicana* that display no lesion growth, infected CD4^+^ T cell specific (Lck^cre^IL-4Rα^−/lox^) IL-4Rα^−/−^ mice initially develop lesions indicating that early susceptibility to *L. mexicana* is dependent on an IL-4 responsive non-CD4^+^ T cell population. However, subsequent lesion growth is significantly curtailed in infected CD4^+^ T cell specific (Lck^cre^IL-4Rα^−/lox^) IL-4Rα^−/−^ mice compared with IL-4Rα intact mice, and a strong Th1 response generated in the presence of significant elements of Th2 activity. Despite reduced susceptibility in all CD4^+^ T cell specific (Lck^cre^IL-4Rα^−/lox^) IL-4Rα^−/−^ mice, a dichotomy between the sexes was identified during *L. mexicana* infection and while lesions in female CD4^+^ T cell specific (Lck^cre^IL-4Rα^−/lox^) IL-4Rα^−/−^ mice healed they persisted in male mice associated with elevated IL-4 production in this sex compared with females. Together, our results suggest that initial development of the *L. mexicana* lesion is dependent on an IL-4/13-responsive non-T cell population, whilst progressive infection is dependent on CD4^+^ T cells responsive to IL-4.

## Methods

### Mouse model

LysM^cre^IL-4Rα^−/−^, Lck^cre^IL-4Rα^−/−^, IL-4Rα^lox/lox^ mice were generated and maintained as previously described [9]–[10]. Cell-specific gene disruption in macrophages/neutrophils or T cells was achieved through an intercross between either LysM^cre^IL-4Rα^−/−^ or Lck^cre^IL-4Rα^−/−^ and IL-4Rα^lox/lox^ mice. Transgene-bearing LysM^Cre^IL-4Rα^−/lox^ and Lck^cre^IL-4Rα^−/lox^, were identified by PCR genotyping as described [9]–[10]. The mice were maintained under specific pathogen free conditions. Animal experiments were performed in strict accordance with the UK Home Office Animal [Scientific Procedures] Act 1986 (licence number 60/3929) with approval by the University of Strathclyde Ethical Review Panel.

### *L. mexicana* parasites and infection

*L. mexicana* (MYNC/BZ/62/M379) was maintained by serial passage of amastigotes inoculated into the shaven rumps of BALB/c mice. Amastigotes for use in infections were isolated from lesions and enumerated as described below. Two sites of infection were examined and either 5×10^6^ *L. mexicana* amastigotes in a final volume of 50µl were inoculated subcutaneously into the shaven base of the tail, or 2×10^5^ *L. mexicana* amastigotes in a final volume of 25µl were inoculated subcutaneously into the hind footpad. 6–8 week old male or female LysM^Cre^IL-4Rα^−/lox^ and Lck^cre^IL-4Rα^−/lox^ mice were used in each experiment, with age and sex matched cre negative IL-4Rα^−/lox^ littermates used as controls. The lesion diameter was measured using a sliding gauge micrometer at weekly intervals.

### Enumeration of parasites

Lesions were excised from *L. mexicana* infected LysM^Cre^IL-4Rα^−/lox^, Lck^cre^IL-4Rα^−/lox^ and IL-4Rα^−/lox^ mice and disrupted through a metal mesh with 5mL of RPMI 1640 (Cambrex Bio Science Verviers, Belgium). The parasites were washed twice at 350g in RPMI and then enumerated using an improved Naubauer haemocytometer. Alternatively parasite numbers were quantified by limiting dilution, as previously described [11].

### Splenocyte stimulation and cytokine detection

Splenocytes were isolated from infected mice and cultured for 72 hours in 96-well plates (Corning-Costar, NY, USA) in the presence or absence of *L. mexicana* antigenic lysate, as previously described [6]. IFN-γ and IL-4 levels were detected in the supernatants by capture ELISA. Briefly the wells of Immulon 1B flat-bottomed microtitre plates (ThermoLabsystems, MA, USA) were coated with 50µL of 1µg ml^−1^ purified anti-mouse IFN-γ capture antibody R4-6A2 (BD Biosciences, Oxford, UK) or 500ng ml^−1^ IL-4 capture antibody 11B11 (BD Biosciences) in PBS (pH 9.0) overnight at 4°C. Supernatants were then added to the individual wells and either 30µL recombinant mouse IFN-γ (R&D Systems, Abingdon, UK) or recombinant mouse IL-4 (Genzyme, Cambridge, UK) added to individual wells in duplicate in a doubling dilution with a solution of pH 7.4 PBS supplemented with 10% v/v FCS (Harlan Sera-Lab Ltd., Crawley, UK), ranging form 20ng mL^−1^ to 0.01ng mL^−1^ (IFN-γ) or 2ng mL^−1^ to 0.977pg mL^−1^ (IL-4). The plates were then incubated for 2 hours at 37°C. The bound cytokines were incubated with either biotinylated rat anti-mouse IFN-γ monoclonal antibody XMG1.2 or biotinylated rat anti-mouse IL-4 antibody BVD6-24G2 (both BD Biosciences) and detected with either conjugated streptavidin-alkaline phosphatase or conjugated streptavidin-horseradish peroxidase (BD Biosciences). The appropriate substrate was then added to the wells, p-nitrophenyl-phosphate (Sigma-Aldrich, Poole, UK) or tetramethylbenzidine in pH 5.5 sodium acetate buffer, containing 0.0003% hydrogen peroxide (BDH, Poole, UK). Finally the plates were read at an absorbance of 405nm for IFN-γ or at 450nm for IL-4.

### Detection of *Leishmania mexicana* specific -IgG1, -IgG2a and total IgE

*L. mexicana* specific-IgG1 and -IgG2a were detected in the plasma of infected mice by ELISA, as previously described [12]. Briefly, Immulon 1B flat-bottomed microtitre plates were coated with 100µL of 10µg ml^−1^ *Leishmania mexicana* lysate (lysate preparation previously described [13] in PBS (pH 9.0) overnight at 4°C. Plasma samples were serially diluted in duplicate, followed by a 1 hour incubation at 37°C. Bound *Leishmania* specific antibodies were detected with a 1 hour incubation with horseradish peroxidase conjugated goat anti-mouse IgG1 or goat anti-mouse IgG2a (Southern Biotechnology Associates Inc., AL, USA). The substrate tetramethylbenzidine in pH 5.5 sodium acetate buffer, containing 0.0003% hydrogen peroxide, was then added to the wells and, following colour development, the reaction stopped by the addition of 10% sulphuric acid, absorbance measured at 450nm using a SOFTmax PRO (Molecular Devices, CA, USA) and the endpoint dilution was determined. Total IgE was detected in the plasma of infected mice by capture ELISA as previously described [12], using R35–72 capture IgE mAb (BD Biosciences) and biotinylated rat anti-mouse IgE (Southern Biotechnology Associates Inc.).

### Flow cytometry

Draining lymph node cells were activated for 4 hours with 50 ng ml^−1^ PMA and 500 ng ml^−1^ Ionomycin (both Sigma-Aldrich) along with GolgiPlug (BD Biosciences). Following stimulation, cells were harvested and washed, resuspended in FACS Buffer containing Fc Block (2.4G2 hybridoma supernatant) together with the appropriate combinations of the following antibodies: CD4-APC, CD8-PerCP or B220-FITC (all from BD Biosciences). Intracellular cytokine staining was carried out using PE-conjugated anti-mouse IL-4 or IFN-γ with Cytofix/Cytoperm solution (all from BD Biosciences). Data was obtained using FACSCanto (BD Bioscience) and analysed using FlowJO (Tree Star Inc., CA, USA).

### Statistical analysis

Antibody analysis was performed using the Mann-Whitney U test and all other analysis used an unpaired Student's t test.

## Results

### IL-4Rα signaling via macrophages/neutrophils plays little role in the susceptibility of BALB/c mice to *L. mexicana*

**Figure 1:**
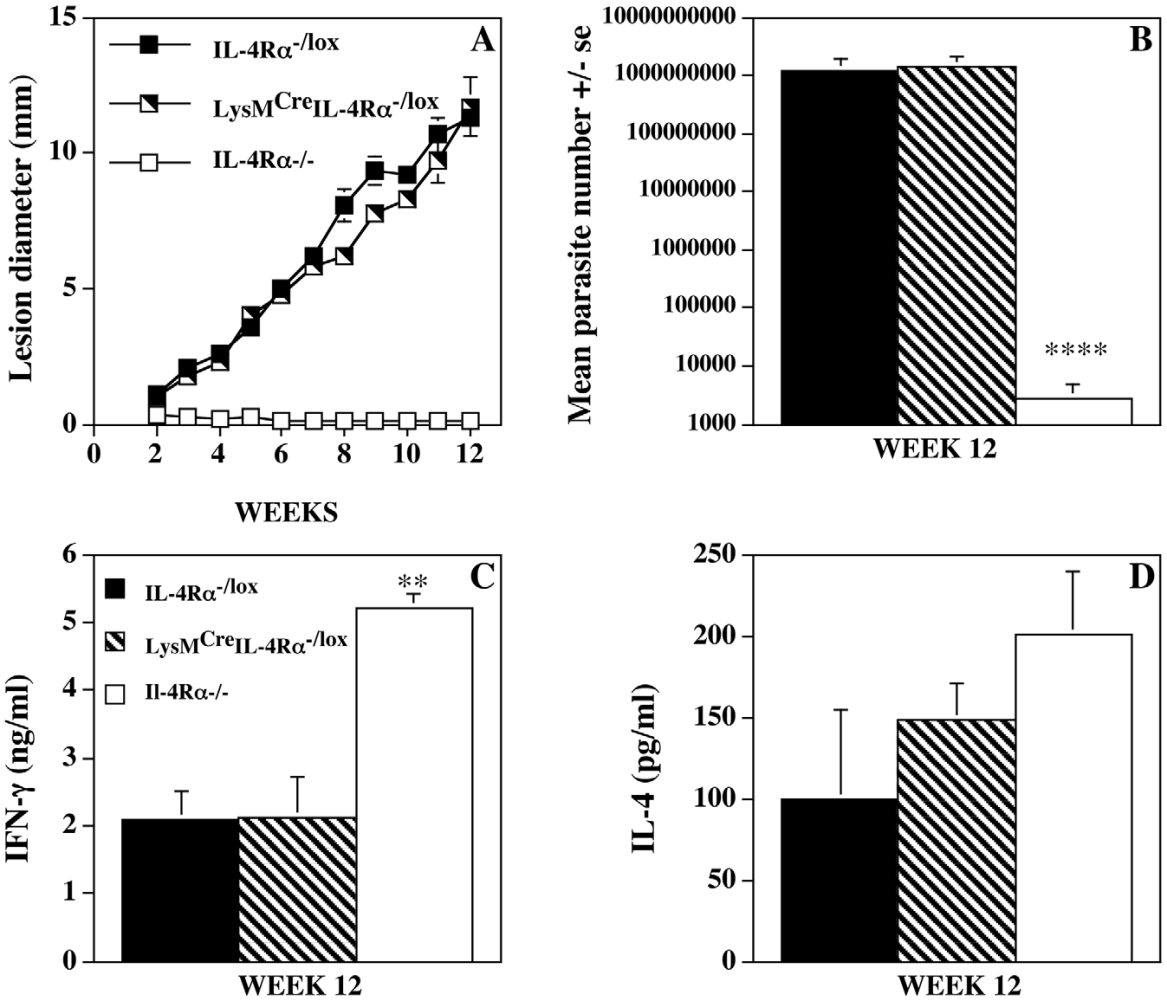
IL-4Rα deficiency in macrophages/neutrophils does not influence *L. mexicana* growth or the immune response. Mean lesion growth (Figure. 1A), parasite burdens (Figure. 1B), and *L. mexicana* antigen induced (25 µg/ml) splenocyte IFN-γ (Figure. 1C), and IL-4 (Figure 1D) produced from IL-4Rα intact (IL-4Rα^−/lox^), macrophage/neutrophil specific (LysM^cre^IL-4Rα^−/lox^) IL-4Rα^−/−^, and global IL-4Rα^−/−^ mice infected sub-cutaneously with 5×10^6^ amastigotes of *L. mexicana*. At 12 weeks post infection parasite burdens were significantly less in IL-4Rα^−/−^ mice than IL-4Rα^−/lox^ or LysM^cre^IL-4Rα^−/lox^ mice (****p<0.0001) and IFN-γ production significantly greater (**p<0.01). Results are mean +/− s.e. 5 mice. Representative of 3 independent experiments.

To compare the progression of *L. mexicana* lesion growth in LysM^Cre^IL-4Rα^−/lox^ with IL-4Rα^−/lox^ littermate control and global IL-4Rα^−/−^ mice, animals were infected with 5×10^6^ amastigotes into the shaven base of the tail. While no discernible lesions were identified in infected global IL-4Rα^−/−^ mice, as previously demonstrated [6], rapidly growing non-healing lesions were observed in both LysM^Cre^IL-4Rα^−/lox^ and IL-4Rα^−/lox^ mice (Figure 1A). Parasite burdens were also similar in LysM^Cre^IL-4Rα^−/lox^, and IL-4Rα^−/lox^ mice and significantly higher (p<0.0001) than those recorded from global IL-4Rα^−/−^ animals (Figure 1B). In line with the non-healing progressive disease phenotype displayed by macrophage/neutrophil IL-4Rα^−/−^ mice, parasite antigen induced spleen cell IFN-γ production was similar to IL-4Rα^−/lox^ mice and significantly less (p<0.01) than that of global IL-4Rα^−/−^ mice (Figure 1C). Antigen induced splenocyte IL-4 production was similar in all 3 strains (Figure 1D), demonstrating once again that IL-4 induction can be independent of IL-4Rα signaling [6], [14]–[16]. Further studies demonstrated that the close similarities in the disease phenotypes of LysM^Cre^IL-4Rα^−/lox^ and IL-4Rα^−/lox^ mice were independent of site, dose of inoculum, and life cycle stage initiating infection (data not shown). These data suggest that the expression of IL-4Rα by a macrophage/neutrophil population is not important in determining susceptibility to *L. mexicana* infection.

### IL-4Rα signaling via CD4^+^ T cells is essential for progressive non-healing disease following infection with *L. mexicana*

**Figure 2:**
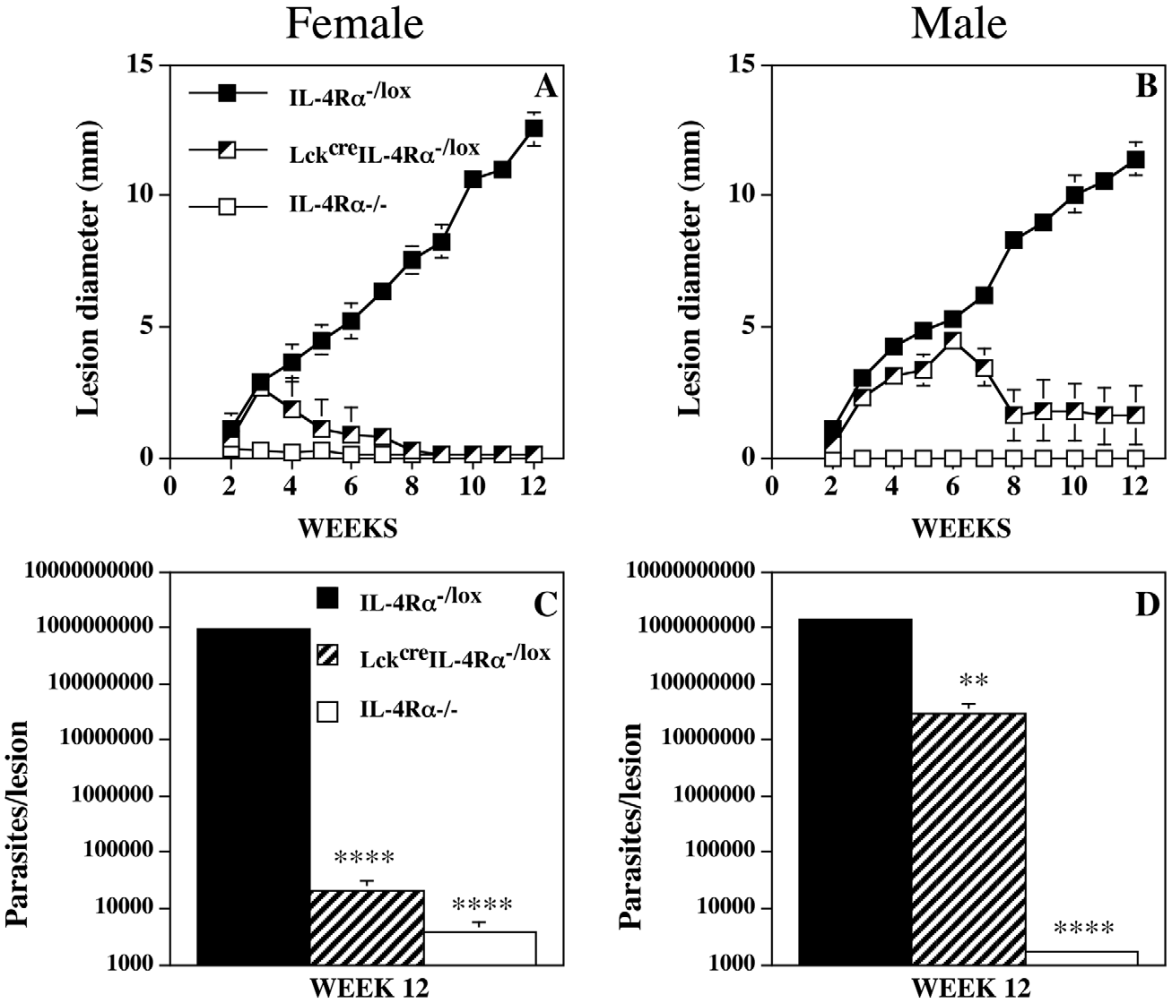
IL-4Rα deficiency in CD4^+^ T cells enables control of *L. mexicana* parasite growth. Mean lesion growth (Figure 2A and B) and parasite burdens at week 12 (Figure 2C and D) in female (Figure 2A and C) and male (Figure 2B and D) IL-4Rα intact (IL-4Rα^−/lox^), CD4^+^ T cell specific (Lck^cre^IL-4Rα^−/lox^) IL-4Rα^−/−^, and global IL-4Rα^−/−^ mice infected sub-cutaneously with 5×10^6^ amastigotes of *L. mexicana*. Results are mean +/− s.e. 5 mice. Representative of 4 separate experiments. Parasite burdens in IL-4Rα^−/−^ male and female mice were significantly less than IL-4Rα intact mice (****p<0.0001) for both male and female mice. Parasite burdens were also significantly less in male and female CD4^+^ T cell specific IL-4Rα^−/−^ compared with IL-4Rα intact mice (****p<0.0001) for females and (**p<0.01) for males. Male CD4^+^ T cell specific IL-4Rα^−/−^ had significantly higher parasite burdens than females on the same background (p<0.001).

In subsequent studies, infection of CD4^+^ T cell specific (Lck^cre^IL-4Rα^−/lox^) IL-4Rα^−/−^ mice with 5×10^6^ *L. mexicana* amastigotes into the shaven base of the tail resulted in control of the lesion growth observed in control IL-4Rα^−/lox^ mice (wild-type equivalent). Interestingly, in one experiment using male mice, lesions in Lck^cre^IL-4Rα^−/lox^ mice did not heal completely, while experiments utilizing female mice fully resolved (Figure S1). Consequently, to confirm and further investigate this apparent gender-dependent difference in control of *L. mexicana* infection male and female Lck^cre^IL-4Rα^−/lox^, control IL-4Rα^−/lox^, and global IL-4Rα^−/−^ mice were infected in parallel with 5×10^6^ *L. mexicana* amastigotes into the shaven base of the tail (Figure 2A–D). While infected global IL-4Rα^−/−^ mice displayed a non-lesion growth phenotype, lesion growth was progressive in control IL-4Rα^−/lox^ mice (Figure 2A and B). These disease phenotypes were independent of gender. By comparison *L. mexicana* infected female CD4^+^ T cell specific (Lck^cre^IL-4Rα^−/lox^) IL-4Rα^−/−^ mice developed lesions which completely healed after 4–5 weeks (Figure 2A), while male CD4^+^ T cell specific (Lck^cre^IL-4Rα^−/lox^) IL-4Rα^−/−^ developed lesions which failed to fully heal (Figure 2B). Indeed in agreement with lesion size parasite burdens up until week 6 were similar in both male Lck^cre^IL-4Rα^−/lox^ and IL-4Rα^−/lox^ mice (Figure S2).

Parasite numbers at the termination of the study at week 12 were of a similar order of magnitude in both female and male control IL-4Rα^−/lox^ mice while global IL-4Rα^−/−^ mice of both sexes were equally able to control infection with *L. mexicana* (Figure 2C and D). By contrast while infected CD4^+^ T cell specific (Lck^cre^IL-4Rα^−/lox^) IL-4Rα^−/−^ mice of both sexes were able to significantly control parasite growth (p<0.0001 and p<0.01 respectively for female and male mice), male mice (Figure 2D) were significantly limited in this ability and had significantly higher parasite burdens (p<0.001) than female mice (Figure 2C).

### IL-4Rα signaling via CD4^+^ T cells inhibits the production of a specific Th1 response following infection with *L. mexicana*

**Figure 3:**
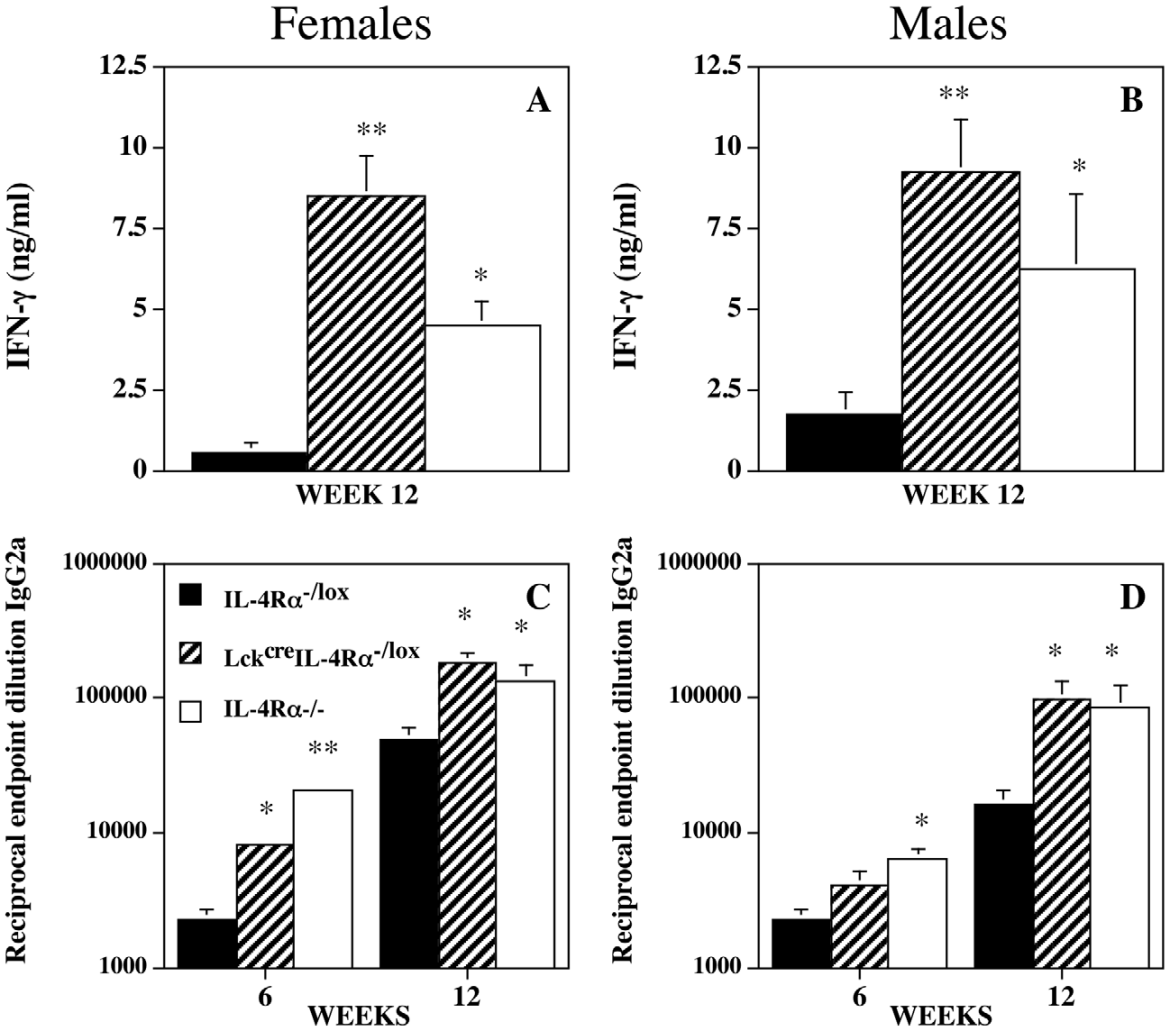
IL-4Rα deficiency in CD4^+^ T cells results in enhanced Th1 responses after *L. mexicana* infection. *L. mexicana* antigen induced (10 µg/ml) splenocyte IFN-γ (Figure 3A and B), and 6 and 12 week serum IgG2a levels (Figure 3C and 3D) produced from female (Figure 3A and C) and male (Figure 3B and D) IL-4Rα intact (IL-4Rα^−/lox^), CD4^+^ T cell specific (Lck^cre^IL-4Rα^−/lox^) IL-4Rα^−/−^, and global IL-4Rα^−/−^ mice infected sub-cutaneously with 5×10^6^ amastigotes of *L. mexicana*. *p<0.05, **p<0.01 compared with IL-4Rα intact mice. Representative of 4 separate experiments.

*L. mexicana* infection of CD4^+^ T cell specific (Lck^cre^IL-4Rα^−/lox^) IL-4Rα^−/−^ mice resulted in an enhanced Th1 response in both male and female Lck^cre^IL-4Rα^−/lox^ mice compared with control IL-4Rα^−/lox^ mice, as demonstrated by significantly enhanced antigen induced splenocyte IFN-γ production (Figure 3A and B; p<0.025 for females; p<0.01 for males). As previously described, antigen stimulated splenocyte IFN-γ production from infected global IL-4Rα^−/−^ mice was significantly higher than for wild-type equivalent (IL-4Rα^−/lox^) mice and this was true whether examining female or male mice (p<0.02). Antigen stimulated splenocytes from infected female but not male Lck^cre^IL-4Rα^−/lox^ mice produced significantly more IFN-γ than antigen stimulated splenocytes from infected global IL-4Rα^−/−^ animals. An expanded Th1 response was also indicated by enhanced antigen specific IgG2a production compared with control IL-4Rα^−/lox^ mice, and in magnitude similar to that generated by global IL-4Rα^−/−^ mice (Figure 3C and D). However, while specific IgG2a production in the absence of IL-4Rα signaling via CD4^+^ T cells in female Lck^cre^IL-4Rα^−/lox^ mice was significantly greater (p<0.025) than in infected control IL-4Rα^−/lox^ mice as early as week 6 post-infection (Figure 3C) it was not until week 12 post-infection that male Lck^cre^IL-4Rα^−/lox^ mice were producing significantly more IgG2a (p<0.025) than their infected control IL-4Rα^−/lox^ counterparts (Figure 3D). Similar results were recorded in 3 separate experiments.

### IL-4Rα signaling via CD4^+^ T cells promotes the induction of a Th2 response following infection with *L. mexicana* differentially in male and female mice

**Figure 4:**
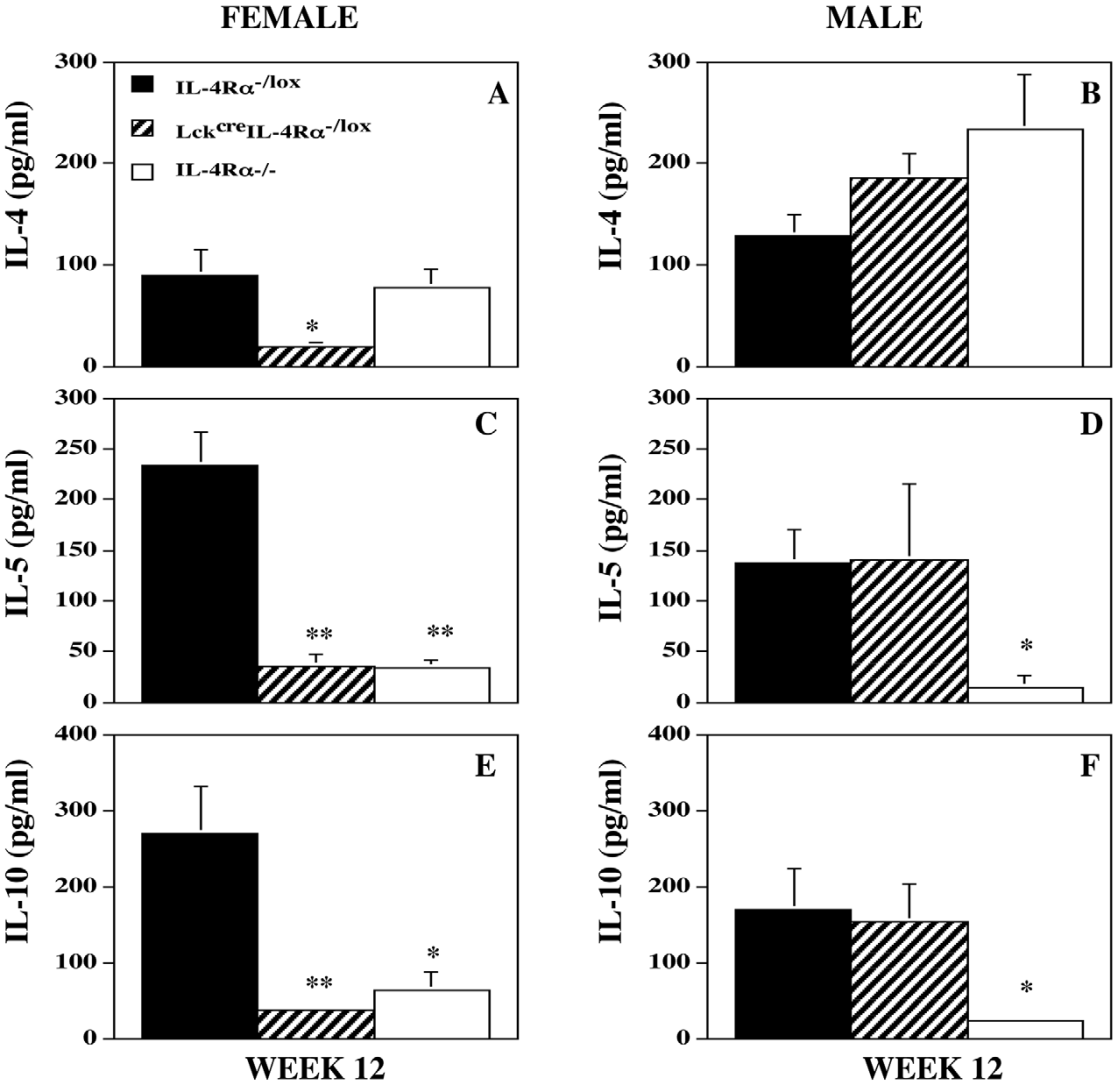
IL-4Rα deficiency in CD4^+^ T cells results in decreased specific Th2 responses in female mice. *L. mexicana* antigen induced (10 µg/ml) splenocyte IL-4 (Figure 4A and B), IL-5 (Figure 4C and D) and IL-10 (Figure 4E and F) produced from female (Figure 4A, C and E) and male (Figure 4B, D and F) IL-4Rα intact (IL-4Rα^−/lox^), CD4^+^ T cell specific (Lck^cre^IL-4Rα^−/lox^) IL-4Rα^−/−^, and global IL-4Rα^−/−^ mice infected sub-cutaneously with 5×10^6^ amastigotes of *L. mexicana*. *p<0.05, **p<0.01 compared with IL-4Rα intact mice. Representative of 4 separate experiments.

A clear dichotomy in antigen-induced splenocyte IL-4 production was identified between infected female and male Lck^cre^IL-4Rα^−/lox^ mice both compared with each other and compared with their wild-type equivalent counterparts (Figure 4A and B). Splenocyte IL-4 production was barely detectable in female Lck^cre^IL-4Rα^−/lox^ mice, and significantly less than IL-4 production by male Lck^cre^IL-4Rα^−/lox^ mice (p<0.01). On the other hand, antigen induced splenocyte IL-4 production was similar in all infected male mice, independent of IL-4Rα expression (Figure 4B). Similar results were obtained with another Th2 cytokine, IL-5 (Figure 4C and D), as well as IL-10 (Figure 4E and F), with production of both cytokines significantly lower in female but not male Lck^cre^IL-4Rα^−/lox^ compared with similarly infected sex matched control IL-4Rα^−/lox^ mice. Similarly, Th2 associated antigen specific IgG1 production was significantly less in infected female (p<0.05 week 12) but not infected male Lck^cre^IL-4Rα^−/lox^ mice compared with their respective control IL-4Rα^−/lox^ counterparts (Figure S2). Minimal IgG1 production was detected in the serum of infected global IL-4Rα^−/−^ mice. Total IgE levels in infected female and male Lck^cre^IL-4Rα^−/lox^ mice were similar to each other and intermediate between those induced in control IL-4Rα^−/lox^ and the absence of IgE in infected IL-4Rα^−/−^ mice (Figure S3).

**Figure 5:**
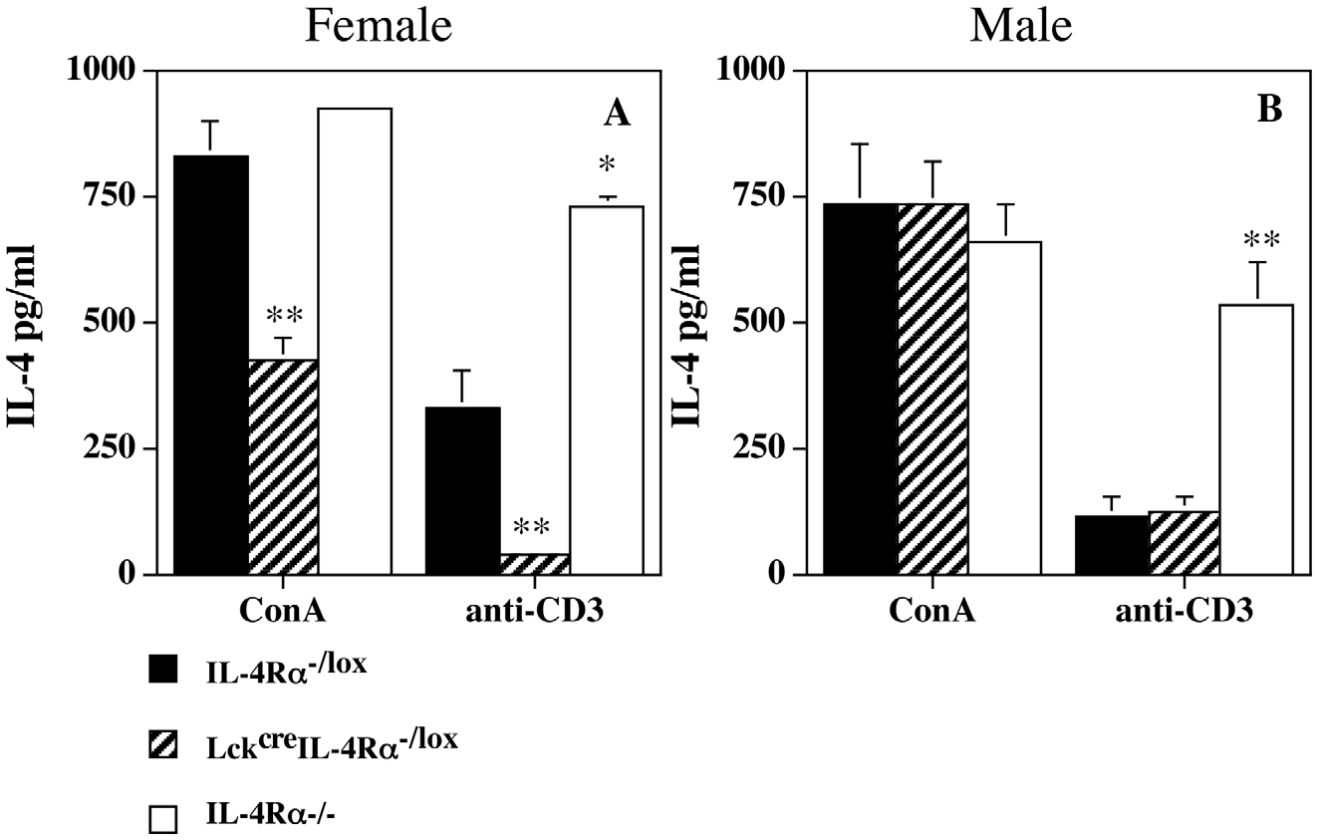
IL-4Rα deficiency in CD4^+^ T cells results in decreased non-specific Th2 responses in female mice. ConA and anti-CD3 induced (10 µg/ml) splenocyte IL-4 produced from female (Figure 5A) and male (Figure 5B) IL-4Rα intact (IL-4Rα^−/lox^), CD4^+^ T cell specific (Lck^cre^IL-4Rα^−/lox^) IL-4Rα^−/−^, and global IL-4Rα^−/−^ mice infected sub-cutaneously with 5×10^6^ amastigotes of *L. mexicana* 12 weeks previously. *p<0.05, **p<0.01 compared with IL-4Rα intact mice. IL-4 production from female Lck^cre^IL-4Rα^−/lox^ mice was significantly less than similarly treated male Lck^cre^IL-4Rα^−/lox^ mice (p<0.02 for ConA and p<0.05 for anti-CD3 respectively). Results are mean+/− s.e. 5 mice. Representative of 2 separate experiments.

**Figure 6:**
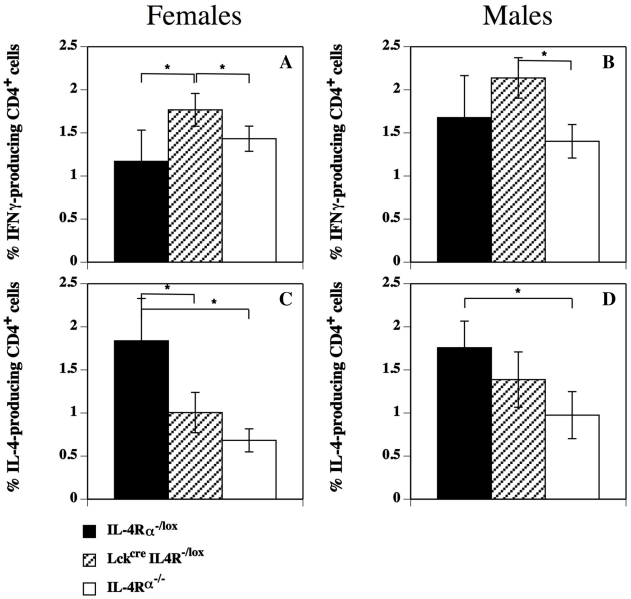
Infected female T cell specific IL-4Rα^−/−^ mice have less IL-4^+^ and more IFN-γ^+^ CD4^+^ cells. Cytokine production by CD4^+^ T cells from draining lymph nodes of *L. mexicana* infected female (Figure 6A and C) and male (Figure 6B and D) IL-4Rα intact (IL-4Rα^/lox^), CD4^+^ T cell specific (Lck^cre^IL-4Rα^−/lox^) IL-4Rα^−/−^, and global IL-4Rα^−/−^ mice as assessed by flow cytometry at 12 weeks post-infection. Results are mean+/− s.d. 5 mice. Representative of 2 separate experiments.

Anti-CD3 and ConA stimulation (Figure 5A and B) of spleen cells from 12 week infected mice demonstrated quite clearly that not only was IL-4 production from female Lck^cre^IL-4Rα^−/lox^ mice significantly less than that of female control IL-4Rα^−/lox^ mice (p<0.003 for ConA and p<0.01 for anti-CD3 respectively), but also significantly less than similarly treated male Lck^cre^IL-4Rα^−/lox^ mice (p<0.02 for ConA and p<0.05 for anti-CD3 respectively). Conversely male Lck^cre^IL-4Rα^−/lox^ mice splenocytes produced similar quantities of IL-4 to control male IL-4Rα^−/lox^ mice with either treatment. Examination of draining inguinal lymph node cells indicated a significantly lower (p<0.05) percentage of IL-4 and greater (p<0.05) percentage of IFN-γ secreting CD4^+^ T cells in infected female but not male Lck^cre^IL-4Rα^−/lox^ mice compared with control sex-matched IL-4Rα^−/lox^ mice (Figure 6 A–D).

## Discussion

We have previously demonstrated that signaling via IL-4Rα plays the major role in the non-healing response of BALB/c mice following infection with *L. mexicana* [6] and that IL-4Rα^−/−^ mice, unlike their wild-type counterparts that produce progressively growing non-healing lesions, display a non-lesion growth disease phenotype associated with an enhanced type-1 response. In the course of the present study using macrophage/neutrophil specific IL-4Rα^−/−^ mice (LysM^cre^IL-4Rα^−/lox^) we failed to identify any significant role for IL-4Rα signaling via macrophages/neutrophils in the normal non-healing response of BALB/c mice infected with *L. mexicana*. In contrast, following early lesion growth, CD4^+^ T cell specific (Lck^cre^IL-4Rα^−/lox^) IL-4Rα^−/−^ mice were able to inhibit disease progression. However, while lesions in female CD4^+^ T cell specific (Lck^cre^IL-4Rα^−/lox^) IL-4Rα^−/−^ mice healed those in male CD4^+^ T cell specific (Lck^cre^IL-4Rα^−/lox^) IL-4Rα^−/−^ mice persisted. Furthermore, although both female and male CD4^+^ T cell specific (Lck^cre^IL-4Rα^−/lox^) IL-4Rα^−/−^ mice had significantly enhanced type-1 responses compared with IL-4Rα intact (IL-4Rα^−/lox^) mice, male CD4^+^ T cell specific (Lck^cre^IL-4Rα^−/lox^) IL-4Rα^−/−^ mice maintained strong type-2 responses compared with their female counterparts.

Although signaling via IL-4Rα plays a significant role in the outcome of infection with *L. mexicana* as well as *L. major* [6], [8]–[9], [15] the cell targets for IL-4/IL-13 activity and whether they promote or inhibit the disease process differ significantly between species. Thus, while IL-4Rα signaling via macrophages/neutrophils promotes early lesion growth in *L. major* infected BALB/c mice and macrophage/neutrophil specific (LysM^cre^IL-4Rα^−/lox^) IL-4Rα^−/−^ mice display delayed lesion growth [8], we have failed to identify any contributory role for IL-4Rα signaling via macrophages/neutrophils during *L. mexicana* infection. The control of *L. major* early in infection in LysM^cre^IL-4Rα^−/lox^ mice has been identified as being due to enhanced macrophage microbicidal NO mediated activity in the absence of alternative macrophage activation. What may be critical in this regard is that *L. amazonensis* parasites, which belong to the “*mexicana*” complex of parasites, have been shown to be more resistant to macrophage-mediated control than *L. major* requiring higher levels of NO to induce killing [16]–[17]. Furthermore, recent evidence indicates that, unlike *L. major*, there is in fact enhanced replication of the amastigote stage of *L. amazonensis* in IFN-γ-stimulated murine macrophages [18], reportedly due to the induction of a novel L-arginine pathway independent of iNOS or host arginase [19]. In addition it has been demonstrated that arginase null-mutant *L. mexicana* have attenuated virulence *in vitro* and *in vivo* with the indication that the parasite arginase has a potential role in depleting host L-arginine available for iNOS activity [20]–[21]. Furthermore, the authors suggest that there could be different roles of arginase between *L. mexicana* and *L. major* as the Th2 response is blunted in animals infected with arginase null mutant *L. mexicana* parasites while pharmacological inhibition of arginase during *L. major* infection did not inhibit the Th2 immune response [22].

CD4^+^ T cell specific (Lck^cre^IL-4Rα^−/lox^) IL-4Rα^−/−^ mice are more resistant than global IL-4Rα^−/−^ mice to infection with *L. major*, indicating that in the absence of a polarized Th2 response, there is a role for an IL-4/IL-13 responsive non-CD4^+^ T cell in early resistance to infection [9]. Conversely CD4^+^ T cell specific (Lck^cre^IL-4Rα^−/lox^) IL-4Rα^−/−^ mice are more susceptible than global IL-4Rα^−/−^ mice to infection with *L. mexicana*, indicating a role for an IL-4/IL-13 responsive non CD4^+^ T cell population in early susceptibility. We have now studied the course of *L. mexicana* infection in newly generated iLck^cre^IL-4Rα^−/lox^ female and male mice that have IL-4Rα deleted on all T cell populations [23]. These produce the same disease and immunological phenotypes as CD4^+^ T cell specific (Lck^cre^IL-4Rα^−/lox^) IL-4Rα^−/−^ mice (data not shown). Consequently IL-4 responsive CD8^+^ T cells do not play a role in early susceptibility or the non-healing response following infection with *L. mexicana*. Studies utilizing macrophage specific BALB/c IL-4Rα^−/−^ mice have demonstrated that IL-4/IL-13 operates through this population to enhance *L. major* parasite growth via alternative macrophage activation [8] and consequently these are unlikely to be the population driving a Th1 response in CD4^+^ T cell specific (Lck^cre^IL-4Rα^−/lox^) IL-4Rα^−/−^ mice. However, IL-4 treatment of BALB/c mice prior to T cell priming has previously been demonstrated to instruct dendritic cells to produce IL-12 and facilitate a protective Th1 response against *L. major* [24] and is required for protective type-1 responses to *Candida* [25]. In addition IL-13 is able to prime monocytes for IL-12 production[26], which is also observed in listeriosis [27]. Furthermore both IL-4 and IL-13 promote CD40L-induced IL-12 production by macrophages and dendritic cells [28]. This would indicate that dendritic cells may be the IL-4/IL-13 responsive cells facilitating protection against *L. major* in the absence of IL-4Rα responsive CD4^+^ T cells in BALB/c mice. As no distinct disease phenotype could be discerned in macrophage/neutrophil specific (LysM^cre^IL-4Rα^−/lox^) IL-4Rα^−/−^ mice compared with IL-4Rα intact animals infected with *L. mexicana*, no IL-4/IL13 responsive non-T cell population can easily be ruled out in promoting early infection against this parasite. While a role for B cells and antibody production in the non-healing response to *L. mexicana* is well established (as reviewed in [29]–[30]), the fact that IL-4Rα^−/−^ BALB/c mice are more resistant to this parasite than IL-4^−/−^ mice suggests that IL-13 responsive cells and consequently non-lymphoid cells via IL-4Rα signaling play a role in disease susceptibility during *L. mexicana* infection [6].

Unlike the epidemiological and experimental reports on *L. major* and *L. tropica* which identify females as more susceptible, females are more resistant than males to cutaneous infection with *L. mexicana* (humans and mice) [5], [12], [31]–[33] and visceral leishmaniasis caused by *L. donovani* (humans) [34]–[35] or *L. infantum* (dogs) [36]. Female DBA/2 mice infected with *L. mexicana* develop much stronger Th1 responses, as measured by IFN-γ production, delayed–type hypersensitivity and IgG2a antibody levels, than similarly infected male mice [12], [32]. Similarly, in humans infected with *L. mexicana*, females generally have increased Th1 responses as measured by DTH reactions and decreased Th2 responses as measured by IgE production than males [33]. The present studies using CD4^+^ T cell specific IL-4Rα^−/−^ BALB/c mice have revealed a previously undetected, underlying male susceptibility to *L. mexicana* involving T cells. Thus, unlike female mice, male mice were unable to resolve infection and overall had a less polarized Th1 response and more polarized Th2 than their female counterparts. This was associated with IL-4 production independently of IL-4Rα signaling in male but not female CD4^+^ T cell specific (Lck^cre^IL-4Rα^−/lox^) IL-4Rα^−/−^ BALB/c mice. The *L. mexicana* induced IL-4 producing Th2 phenotype in male Lck^cre^IL-4Rα^−/lox^ BALB/c mice was not the result of differential Cre-mediated deletion efficiency of IL-4Rα in male mice as compared with female mice, as no CD4^+^ T cell population expressing IL-4Rα was detected from either gender (data not shown). While IL-4 production independently of IL-4Rα signaling has been observed in a number of immunological models previously [6], [14]–[15] this is the first time a sex associated influence on this ability has been identified. How significant this could be with regard to the numerous gender related differences observed in inflammatory and infectious diseases is an intriguing question but outside the scope of the present study.

To conclude in this study utilising tissue specific IL-4Rα^−/−^ mice we demonstrate that upon infection with *L. mexicana* initial lesion growth is dependent on a non-CD4^+^ T cell population responsive to IL-4/IL-13, while progressive infection is dependent on CD4^+^ T cells responsive to IL-4. Furthermore whether lesions heal or not is gender determined, suggesting a subtle but significant effect of sex hormones on CD4^+^ T cell function whereby infected male but not female CD4^+^ T cell specific IL-4Rα^−/−^ mice can drive IL-4 production independently of IL-4Rα signaling.

## Supporting Information

Figure S1 Female but not male T cell specific IL-4Rα^−/−^ mice heal following *L. mexicana* infection. Mean lesion growth (Figure 1A and B) in female (Figure 1A) and male (Figure 1B) IL-4Rα intact (IL-4Rα^−/lox^), CD4^+^ T cell specific (Lck^cre^IL-4Rα^−/lox^) IL-4Rα^−/−^, and global IL-4Rα^−/−^ mice infected sub-cutaneously with 5×10^6^ amastigotes of *L. mexicana*. These are the results from 2 separate experiments carried out at different times the first using females (Figure 1A), the second using males (Figure 1B). While lesions healed in female CD4^+^ T cell specific (Lck^cre^IL-4Rα^−/lox^) mice they persisted in male CD4^+^ T cell specific (Lck^cre^IL-4Rα^−/lox^). Results are mean+/− s.e. Additional experiments utilised male and female groups infected in parallel.
(0.20 MB TIF)

Figure S2 Similar parasite burdens in wild-type and T cell specific IL-4Rα^−/−^ male mice 6 weeks post-infection. Mean lesion parasite burden ± s.e. at week 6 post-infection in male IL-4Rα intact (IL-4Rα^−/lox^), and CD4^+^ T cell specific (Lck^cre^IL-4Rα^−/lox^) IL-4Rα^−/−^mice infected sub-cutaneously with 5×10^6^ amastigotes of *L. mexicana*.
(0.11 MB TIF)

Figure S3 Less IgG1 production in infected female T cell specific IL-4Rα^−/−^ mice. *L. mexicana*-specific IgG1 levels (Figure. 2A and B) and total IgE levels (Figure. 2C and D) in female (Figure 2A,and C) and male (Figure 2B, and D) IL-4Rα intact (IL-4Rα^−/lox^), CD4^+^ T cell specific (Lck^cre^IL-4Rα^−/lox^) IL-4Rα^−/−^, and global IL-4Rα^−/−^ mice infected sub-cutaneously with 5×10^6^ amastigotes of *L. mexicana*. * p<0.05, and ***p<0.001 compared with IL-4Rα intact mice. Representative of 4 separate experiments.
(0.38 MB TIF)
