# Supplementary figures and images for: BALB/c Mice Deficient in CD4+ T Cell IL-4Rα Expression Control Leishmania mexicana Load although Female but Not Male Mice Develop a Healer Phenotype
Source: PLoS Negl Trop Dis. 2011 Jan 4;5(1):e930. doi: 10.1371/journal.pntd.0000930 (PMC3014948; doi:10.1371/journal.pntd.0000930)

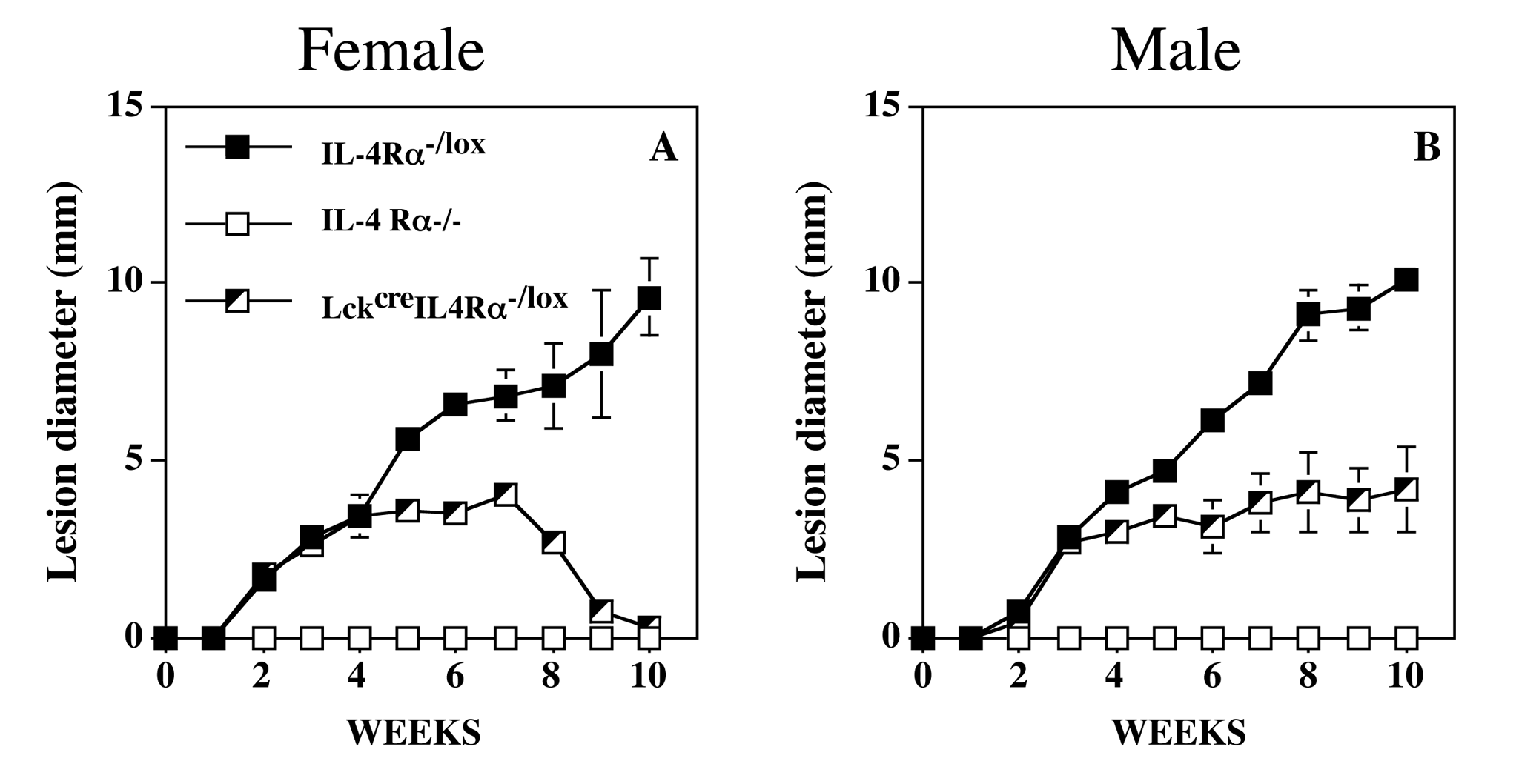

Supplement: Figure S1 — Female but not male T cell specific IL-4Rα−/− mice heal following L. mexicana infection. Mean lesion growth (Figure 1A and B) in female (Figure 1A) and male (Figure 1B) IL-4Rα intact (IL-4Rα−/lox), CD4+ T cell specific (LckcreIL-4Rα−/lox) IL-4Rα−/−, and global IL-4Rα−/− mice infected sub-cutaneously with 5×10^6^ amastigotes of L. mexicana. These are the results from 2 separate experiments carried out at different times the first using females (Figure 1A), the second using males (Figure 1B). While lesions healed in female CD4+ T cell specific (LckcreIL-4Rα−/lox) mice they persisted in male CD4+ T cell specific (LckcreIL-4Rα−/lox). Results are mean+/− s.e. Additional experiments utilised male and female groups infected in parallel. (0.20 MB TIF) [file pntd.0000930.s001.tif]

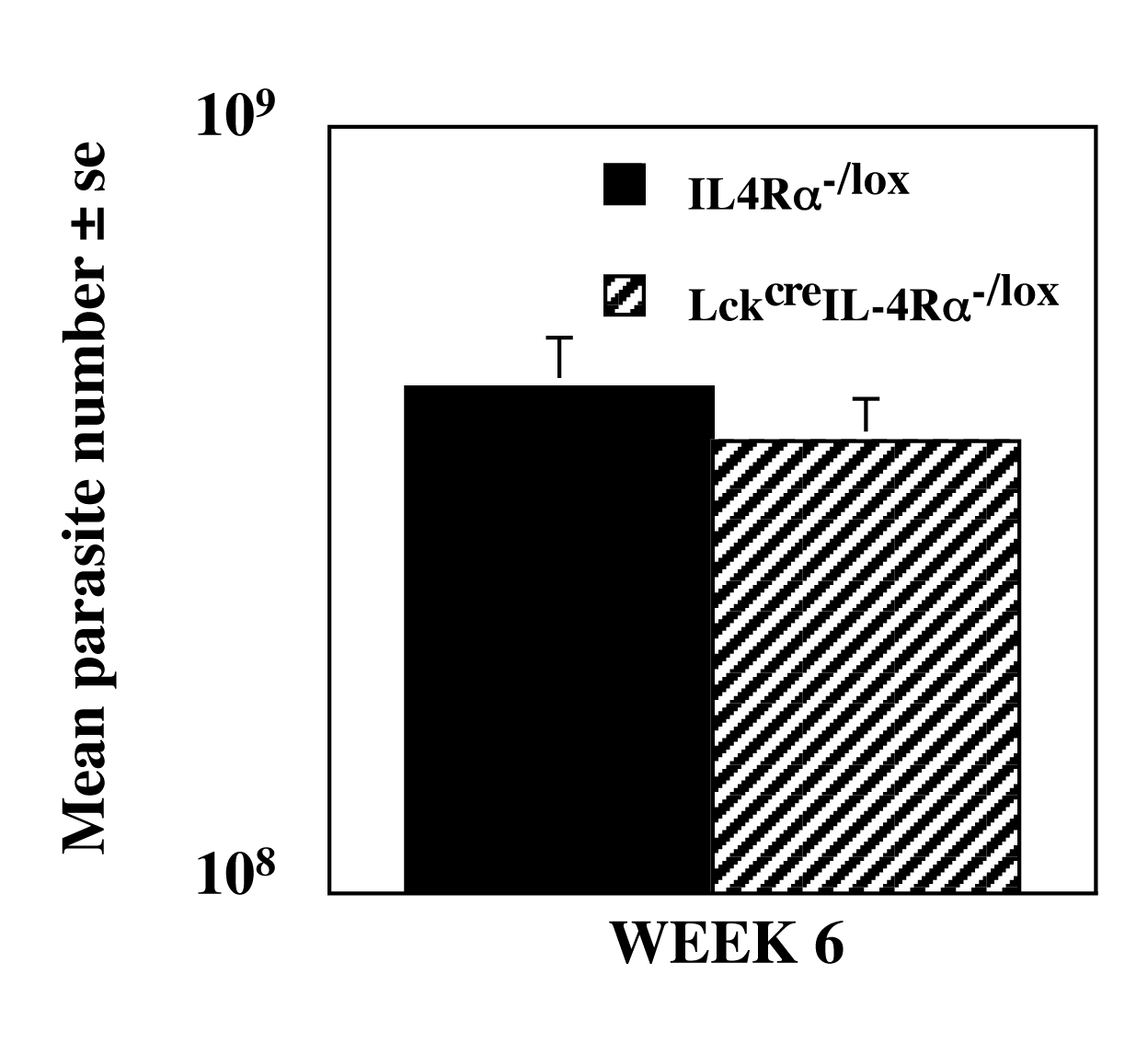

Supplement: Figure S2 — Similar parasite burdens in wild-type and T cell specific IL-4Rα−/− male mice 6 weeks post-infection. Mean lesion parasite burden ± s.e. at week 6 post-infection in male IL-4Rα intact (IL-4Rα−/lox), and CD4+ T cell specific (LckcreIL-4Rα−/lox) IL-4Rα−/−mice infected sub-cutaneously with 5×10^6^ amastigotes of L. mexicana. (0.11 MB TIF) [file pntd.0000930.s002.tif]

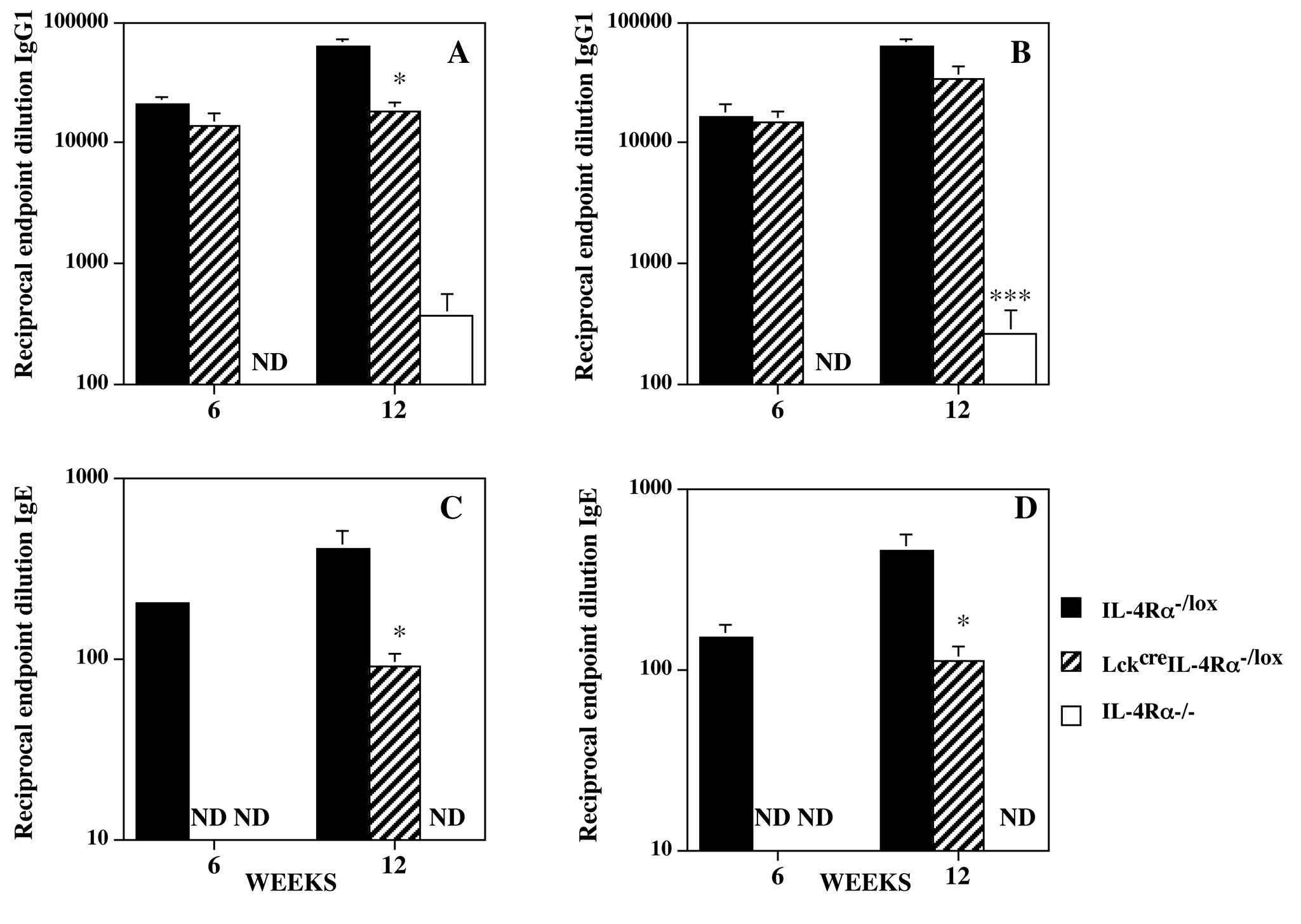

Supplement: Figure S3 — Less IgG1 production in infected female T cell specific IL-4Rα−/− mice. L. mexicana-specific IgG1 levels (Figure. 2A and B) and total IgE levels (Figure. 2C and D) in female (Figure 2A,and C) and male (Figure 2B, and D) IL-4Rα intact (IL-4Rα−/lox), CD4+ T cell specific (LckcreIL-4Rα−/lox) IL-4Rα−/−, and global IL-4Rα−/− mice infected sub-cutaneously with 5×10^6^ amastigotes of L. mexicana. * p<0.05, and ***p<0.001 compared with IL-4Rα intact mice. Representative of 4 separate experiments. (0.38 MB TIF) [file pntd.0000930.s003.tif]
